# The role of information search and its influence on risk preferences

**DOI:** 10.1007/s11238-017-9623-y

**Published:** 2017-07-06

**Authors:** Orestis Kopsacheilis

**Affiliations:** School of Economics, University of Nottingham, Sir Clive Granger Building, Nottingham, NG7 2RD UK

**Keywords:** Decisions from experience, Decisions from description, Risk preferences, Cumulative prospect theory, Uncertainty, Source method, Information search

## Abstract

According to the ‘Description–Experience gap’ (DE gap), when people are provided with the descriptions of risky prospects they make choices as if they overweight the probability of rare events; but when making decisions from experience after exploring the prospects’ properties, they behave as if they underweight such probability. This study revisits this discrepancy while focusing on information-search in decisions from experience. We report findings from a lab-experiment with three treatments: a standard version of decisions from description and two versions of decisions from experience: with and without a ‘history table’ recording previously sampled events. We find that people sample more from lotteries with rarer events. The history table proved influential; in its absence search is more responsive to cues such as a lottery’s variance while in its presence the cue that stands out is the table’s maximum capacity. Our analysis of risky choices captures a significant DE gap which is mitigated by the presence of the history table. We elicit probability weighting functions at the individual level and report that subjects overweight rare events in experience but less so than in description. Finally, we report a measure that allows us to compare the type of DE gap found in studies using choice patterns with that inferred through valuation and find that the phenomenon is similar but not identical across the two methods.

## Introduction

Uncertainty pervades almost every sphere of economic activity and understanding and predicting the choices people make under uncertain circumstances has been a central goal for decision theorists. Among the plethora of theories of risky behaviour, Cumulative Prospect Theory (henceforth CPT; Tversky and Kahneman 1992) has emerged as the descriptive benchmark for laboratory experiments where lotteries’ properties (list of all possible outcomes and associated probabilities) are fully described (Barberis 2013). One of its key tenets is the claim that people tend to overweight low probability events. However, outside of the laboratory people do not often have access to such explicit numerical summaries of uncertainty.

To study more naturalistic situations, psychologists have recently revived the concept of ‘Decisions From Experience’ (DFE). Within this programme, the ‘sampling paradigm’ (Hertwig et al. 2004) has emerged as the most common lab-implementation of DFE. Unlike ‘Decisions From Description’ (DFD) where the properties of lotteries are explicitly described, subjects in DFE have to explore risky options by sampling from their content (in a computerised setting) prior to making a decision. On each screen, there are typically two such options, each with up to two different possible outcomes. Subjects can experience these outcomes and their relative frequency by clicking on each option. Sampling helps subjects decide which option they want to draw from in a final trial involving real monetary consequences. Unlike this final trial, none of the draws during sampling has any monetary effect. Comparing choices between DFD and DFE, a consistent discrepancy has emerged: in DFD—and in accord with CPT’s tenets—people make choices as if they *overweight* rare^1^ events; whereas, in DFE, it is as if they *underweight* such events (Hertwig et al. 2004).

Several studies (e.g. Hau et al. 2008; Ungemach et al. 2009) have since replicated and explored the underpinnings of the ‘Description–Experience gap’ (DE gap), offering both a wealth of insights and some important open questions (see de Palma et al. 2014 for a recent review). In this study, we address some of those questions by conducting a laboratory experiment with three treatments: a standard version of DFD and two variations of DFE. Our contribution to this literature is threefold.

*First*, we look at sampling patterns in DFE. One of the earliest and most robust findings is that subjects typically rely on small samples where rare events tend to be under-represent (Hertwig 2012). We investigate how people adjust their search strategy as a function of the rarity of an event by looking at the correlation of sampling amount and a lottery’s variance: low variance lotteries in our context contain rarer events.

Lejarraga et al. (2012) study a similar concept, that of “experienced outcome variability” which occurs when a subject samples more than one outcome from a given option. The authors find that this variability correlates with higher levels of sampling and conclude that people are motivated to sample more from lotteries for which they have experienced more than one outcome. Mehlhorn et al. (2014), however, question the direction of this causality by pointing to an endogeneity concern: the likelihood of observing more than one outcome increases with the sampling amount. It is, therefore, possible that high levels of sampling are causing subjects to experience more than one outcome rather than the other way around and conclude that the driver of search effort is ‘anticipated’ rather than ‘experienced’ outcome variability. Studying the relationship between sampling amount and variance contributes to this dialogue in the following way. First, variance is a structural property of the lottery and, therefore, unlike experienced variability, remains unaffected by sampling amount. Moreover, high variance causes variability: a subject is more likely to experience more than one outcome from a ‘50–50’ rather than from a ‘99–1’ distribution. Therefore, if Lejarraga et al.’s thesis holds true, we would expect search effort to correlate positively both with experienced outcome variability and with variance. If, however, sampling amount is positively correlated with variability but negatively correlated with variance, the evidence would favour Melhorn et al.’s objection. Given the relation of variance with rare events, this is equivalent to asking whether subjects sample more from lotteries with rarer events.

Another key novelty of our design is the introduction of a history table in one of our DFE variations: DFE-HT. This table records sampled events and displays them to subjects when they later evaluate the lottery. We examine how its presence influences search by comparing DFE-HT with a more standard version of DFE, DFE-NoHT where there is no such record.^2^ One of the reasons we include this table relates to the role of memory constraints. If subjects rely significantly on memorisation during sampling then the history table will help them alleviate part of the associated cognitive load. If this is the case, we would expect to observe larger samples in DFE-HT than in DFE-NoHT. Because the role of memory is elusive to pinpoint (Wulff et al. 2016) we tackle it from two additional angles: by including a test of working memory and by examining whether sampling undertaken just before the moment of decision has more impact than sampling undertaken earlier (‘recency effect’).

*Second*, we search for potential differences on revealed preferences between these three ways of acquiring information: from description and from autonomous sampling with or without a history table. We record these preferences via a method of repeated choices between a risky and a safe option (see bisection method under 2.1). By comparing choice patterns across these three treatments we examine whether there is a DE gap in our data and if so, whether it is amplified or mitigated by the presence of the history table. Moreover, we elicit CPT’s components (in the gains domain only) at the individual level. For this we rely on the methodology introduced by Abdellaoui et al. (2011b), henceforth AHP, who recently applied the ‘source method’ (Tversky and Fox 1995; Abdellaoui et al. 2011a, b) to study this gap. This method maps different sources of uncertainty (such as DFD and DFE) onto distinct probability weighting functions (weighting functions for short). By examining the shape of the elicited aggregate weighting functions we revisit an interesting tension in this literature: if subjects really underweight in DFE then CPT would prescribe a S-shaped weighting function as opposed to the standard inverse S-shaped curve assigned to DFD. We refer to this potential contrast between the weighting functions in DFD and DFE as the ‘underweighting hypothesis’.

Recent papers were unsuccessful in validating this pattern. AHP for example report that CPT’s standard inverse S-shaped weighting function fits both DFD and DFE well and find that the aggregate weighting function in DFE lies systematically below the function elicited in DFD. They attribute this pattern to a reduced willingness to bet in DFE which is induced by ambiguity aversion: subjects in DFE are less confident about the properties of the sampled options than subjects in DFD. We will refer to this pattern of the DE gap where both weighting functions are inverse-S shaped but that of DFE lies beneath that of DFD as the ‘ambiguity aversion hypothesis’.

An attractive feature of AHP’s methodology is that it allows the elicitation of decision weighting functions at the individual level both parametrically and non parametrically. Additionally, this elicitation permits the manipulation of the degree and precision of the elicited curve. We follow this method and address the tension between the two hypotheses regarding the shape of weighting curves. Suspecting that rare events may hold the key to this investigation, we build on AHP’s method by eliciting significantly more observations in the neighbourhood of rare events.

*Third*, we address an important methodological question that derives from AHP’s adaptations of the sampling paradigm. There are four noticeable differences between the two approaches. First, if an event is never experienced in the sampling paradigm the subject is likely to remain ignorant about its existence. This is not the case with the AHP method where the list of outcomes is always eventually presented to the subject. Second, in the sampling paradigm sampled events reveal corresponding pecuniary outcomes. In contrast, sampled events are represented by different pairs of colours in the AHP method which are only later associated with monetary outcomes. Third, in the sampling paradigm subjects sample from two options at a time while in AHP only from one. Fourth and perhaps most importantly, there is a sharp distinction between the ways the two methods infer the DE gap. In the sampling paradigm this is done by comparing frequencies with which riskier options are chosen over safer ones between DFD and DFE. This comparison does not need to assume a preference model. In contrast, AHP elicit certainty equivalents (CEs), which are prices that make subjects indifferent between keeping or trading the lottery being evaluated. CEs are then used to estimate CPT’s weighting functions and the DE gap is inferred by comparing their shape between DFD and DFE.

These differences raise the question of whether the sampling paradigm’s DE gap is qualitatively similar to that reported by AHP or perhaps a different phenomenon altogether. We take a first step in answering this question by identifying the key DE gap properties inferred through choice proportion comparisons. We then examine how well these properties replicate under our valuation framework which is similar to the one AHP used to infer the DE gap in weighting. We do so by exploiting a feature of AHP’s implementation of the bisection method: a hybrid between valuation and choice methods that elicits CEs by repeated choices between a risky and a safe option.

In what follows, Sect. 2 describes in detail our experimental and elicitation methods. Section 3 presents the ensuing results and Sect. 4 discusses their implications. Finally Sect. 5 concludes.

## Methods

### Experimental design

We conduct a laboratory experiment with three treatments using a between-subjects protocol.^3^ These treatments are: a standard version of DFD and two variations of DFE, DFE-NoHT and DFE-HT.

Treatments consist of 19 time periods and in each period subjects evaluate a lottery. These lotteries are represented by virtual decks of cards, each containing two types of cards demarcated by different pairs of colours. In each period subjects first learn about the relative frequency of each colour. These colours are then linked with monetary outcomes and subjects are asked to evaluate the corresponding lottery.

**Fig. 1:**
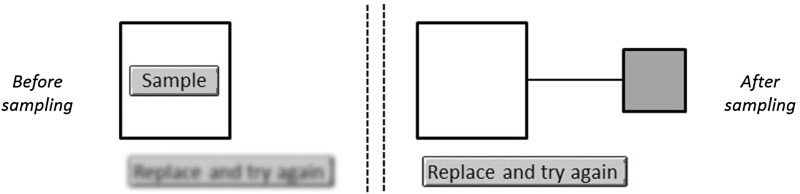
Sampling stage. Screen before (*left*) and after (*right*) a card is drawn. After drawing a card and seeing its *colour*, subjects can replace it in the deck where it gets re-shuffled. They can repeat this for as long as they want. This sampling process is identical in DFE-NoHT and DFE-HT and it appears on a separate screen from the evaluation part. Unlike most sampling technologies, there was no time delay between two consecutive draws. Subjects regulate the time the card remains on screen by pressing on the ‘*replace*’ and ‘*sample*’ buttons at their own discretion

The key difference between DFD and the two DFE treatments lies in the way subjects learn about these relative frequencies. In *DFD* subjects are informed via numerical descriptions, framed as one shot probabilities (E.g. ‘90% of the cards are blue and 10% are red’; see Appendix 6.1 for an instance of this). In contrast, both DFE-treatments require that subjects find out about these likelihoods by sampling colours from the content of the deck in a separate sampling stage (Fig. 1).

The first 7 periods correspond to lotteries with the same probability distribution (but differing outcomes). To communicate this, subjects in DFE go through only one sampling stage, linked to 7 evaluation parts. Therefore, there were only 13 sampling stages in total in DFE. Lotteries and colour-pairs are randomized for each subject across periods. The first 7 lotteries are randomized only within that first cluster.

**Fig. 2:**
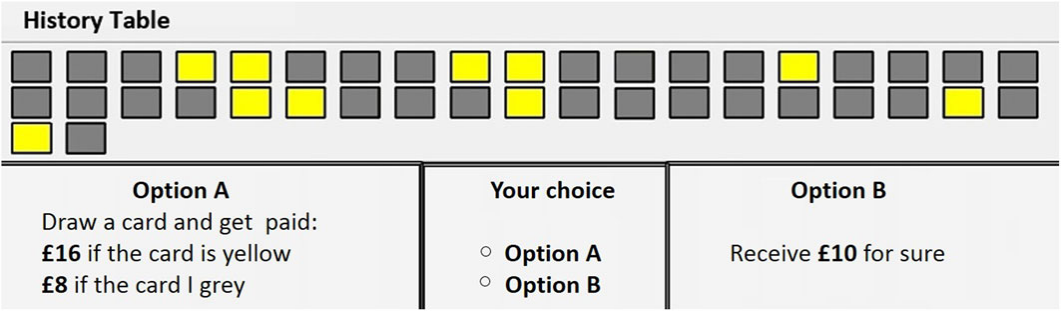
Evaluation part in *DFE-HT*. Sampled events from the sampling stage are recorded and dispayed on the *top* of the evaluation screen in DFE-HT. This part of the screen remained blank in DFE-NoHT

The only difference between the two DFE treatments is the presence (or absence) of the history table during the evaluation part. After subjects in DFE-HT finish sampling and proceed to the next screen associated with the evaluation part, they see a table that has recorded the colours of cards they encountered during sampling, in the order they saw them (see Fig. 2). This history table could only record up to a fixed number of cards. When during sampling this capacity was reached, a message appeared on screen informing subjects that they can continue sampling should they want to, but that their observations past this point would not be recorded. We chose a maximum capacity of 57 with the intention of avoiding a straight-forward calculation of a relative frequency in numeric form, resembling the information in DFD.

The evaluation protocol is common for all three treatments. In this section events (such as ‘Drawing a yellow card’) are associated with monetary consequences. We use the bisection method as applied by AHP to elicit CEs for each lottery. An instance of this can be seen at the bottom of Fig. 2. Every bisection process starts with a choice between a lottery and its expected value offered with certainty. Lotteries are presented under Option A while certain amounts under Option B. The method proceeds by updating Option B until a value close to indifference is reached. In our experiment there were 5 such iterations for each lottery. In Fig. 2’s example, if the subject chooses Option B then the certain outcome will be updated to £9, the midpoint between the lowest outcome of the lottery and the certain outcome that was just chosen. If instead Option A is selected, then Option B will be updated to £13, the midpoint between the highest outcome of the lottery and the certain outcome that was just rejected.

This elicitation through iterative one-shot choices makes the bisection robust against the criticism that methods such as the multiple price list have received (see Erev et al. 2008 for such a criticism). Most importantly for our analysis is the fact that the very first choice in each new evaluation is always between a lottery and a monetary outcome of equivalent expected value (EV) offered with certainty. This is much like the setup that studies in the sampling paradigm have used to infer the DE gap in choice.

Finally, after all lotteries are evaluated, subjects go through a standard forward digit span task where they are asked to recall sequences of digits. Reporting correctly a digit awards the participant a point^4^ and increases the sequence by one digit. After three errors the process is terminated. We use this task as a proxy for memory capacity.

Sessions were conducted in CeDEx’s laboratory at the University of Nottingham. All treatments were programmed in z-Tree (Fischbacher 2007). In total, 118 subjects took part in only one of these three treatments: 40 in* DFE-HT*, 39 *DFE-NoHT* and 39 in *DFD*. We used ORSEE (Greiner 2004) for the recruitment process. At the end of the experiment one question was randomly selected and each subject would get paid according to their choice in that question. Average payment was £11, including a £3 participation fee, for approximately 1-h sessions.

### Elicitation of CPT in DFD and DFE

#### Preliminaries

Let $x_{E_\mathrm{p}}y$ stand for a binary lottery where *x*, *y* are non-negative outcomes^5^ contingent on mutually exclusive events and $x>y$. $E_\mathrm{p}$ represents an event occuring with objective probability *p* and the high (or desirable) outcome *x* is always contingent to $E_\mathrm{p}$. According to CPT, given a strictly increasing utility function: *u* and a weighting function *W*, subjects maximize:

1 $$\begin{aligned} x_{E_\mathrm{p}} y \mapsto W(E_\mathrm{p}) u(x) + (1-W(E_\mathrm{p})) u(y) \end{aligned}$$

To make (1) operational we use the two-stage model idea proposed by Tversky and Fox (1995) and later developed into the ‘source method’ by Abdellaoui et al. (2011a, b). According to this model a decision maker first forms a subjective belief for an uncertain event ($P(E_\mathrm{p})$) and then transforms this value into willingness to bet via a probability weighting function:

2 $$\begin{aligned} W(E_\mathrm{p})= w_\sigma (P(E_\mathrm{p})) \end{aligned}$$

In (2), $w_\sigma (\cdot )$ is the probability weighting function which depends on $\sigma$, the source of uncertainty. Applying (2) to (1) we get:

3 $$\begin{aligned} x_{E_\mathrm{p}} y \mapsto w_\sigma (P(E_\mathrm{p})) u(x) + (1-w_\sigma (P(E_\mathrm{p}))) u(y) \end{aligned}$$

We can break down (3) into: (1) utility over monetary outcomes, $u(\cdot )$, (2) probability measure over outcome distribution, $P(\cdot )$ and (3) source-dependent probability weighting function, $w_\sigma (\cdot )$. The source method adjusts this third component according to the environment where the risky choice takes place.

In DFD we are in an environment where probabilities are completely known and so $p=E_\mathrm{p}$. When analysing DFE on the other hand, we are referring to an environment where probabilities cannot be calculated exactly but can instead be assessed in an empirical manner by the subject. To apply (3) in DFE, given that the belief $P(E_\mathrm{p})$ is essentially unobservable, we consider the following two proxies: Objective (or true) probability (*p*) and experienced probability ($f_\mathrm{p}$). The latter stands for the relative frequency with which an event has been observed in a sample.

Using true probabilities as proxies for beliefs, although convenient and widely used in this literature, can be problematic—especially in cases where sampling bias is prevalent. Therefore, our analysis proceeds by reporting (mostly) experienced probabilities. Although this proxy might still not be perfect, there has been evidence for a high correlation between elicited beliefs and $f_\mathrm{p}$ (Fox and Hadar 2006).

#### Estimation

Our approach is based on AHP’s adaptation of the semi-parametric method developed by Abdellaoui et al. (2008). We use 16 lotteries (Table 1; lotteries 1–16) which we separate into two clusters. In the first cluster subjects evaluate 7 lotteries with a fixed probability $p=0.25$. The reported CEs are then used for the estimation of a utility function. Assuming the power-function specification: $u(x)=x^\alpha$, we need only estimate $(W(E_{0.25}), \alpha )$ for each subject, where $\alpha$ captures the curvature of the utility function and $W(E_{0.25})$ the weight assigned to $E_{0.25}$. We do so by minimizing the non-linear least square function: $\Vert z-{\hat{z}} \Vert ^2$, where $z_i$ refers to the observed CE and ${\hat{z}}_i$:

4 $$\begin{aligned} {\hat{z}}_i=\left[ W\left( E_{0.25}\right) \left( x_i^\alpha -y_i^\alpha \right) +y_i^\alpha \right] ^{\frac{1}{\alpha }} \end{aligned}$$

**Table 1:** Lotteries

	Utility							Decision weights									Control		
	1	2	3	4	5	6	7	8	9	10	11	12	13	14	15	16	17	18	19
*x*	4	8	12	16	16	16	16	16	16	16	16	16	16	16	16	16	3	4	4
*p*	0.25	0.25	0.25	0.25	0.25	0.25	0.25	0.025	0.05	0.10	0.25	0.50	0.75	0.90	0.95	0.975	0.25	0.20	0.80
*y*	0	0	0	0	4	8	12	0	0	0	0	0	0	0	0	0	0	0	0

Lotteries 1–7 were used to estimate the utility function while lotteries 8–16 to elicit weighting functions (both parametrically and non-parametrically). Lotteries 17–19 are not relevant for the estimation and were included as control tasks due to their similarity with some of the commonly used lotteries in the early DE gap studies

In the second cluster subjects evaluate a total of 9 lotteries with fixed high ($x=\pounds 16$) and low ($y=\pounds 0$) outcomes and varying *p*. Subsequently, using the estimated $\alpha$ from the first cluster of lotteries, we can control for risk curvature and calculate non-parametrically decision weights^6^ for each level of *p*.

Let $z'_j$ stand for the observed CE elicited from this second cluster of lotteries. Then from (4) we get that:

5 $$\begin{aligned} W(E_{p_j})= \Big (\frac{z'_j}{16}\Big )^{\alpha } ,\quad \mathrm{{for}}\,\, j=1,\ldots ,9 \end{aligned}$$

Finally, we used these decision weights to fit the following two-parameter, linear-in-log-odds weighting function introduced by Goldstein and Einhorn (1987).

6 $$\begin{aligned} w(p)=\frac{\delta p^\gamma }{\delta p^\gamma + (1-p)^\gamma } \end{aligned}$$

This is the same weighting function that AHP used. Parameter $\gamma$ controls curvature with $\gamma <1$ indicating an inverse S-shaped weighting function while $\gamma >1$ a S-shaped one (values close to 1 point to no curvature). Parameter $\delta$ controls elevation with $\delta <1$, $\delta >1$ and $\delta =1$ pointing to ‘low’, ‘high’ and ‘no’ elevation, respectively. Gonzalez and Wu (1999) an interesting psychophysical interpretation for these parameters according to which $\gamma$ is interpreted as a measure of probabilistic sophistication while $\delta$ as a degree of optimism.

## Results

### Sampling

**Fig. 3:**
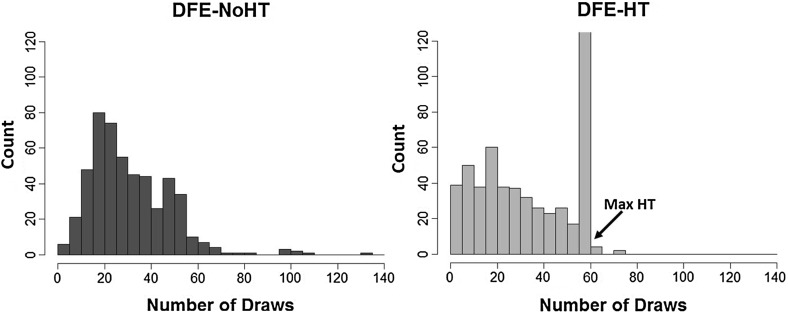
Distribution of draws across DFE-treatments. ‘Max HT’ points to the maximum capacity of the history table (57 draws). Subjects could sample past that point but their observations would not be recorded in the history table

We start by comparing sampling patterns between the two DFE treatments. Figure 3 foreshadows the importance of the history table in influencing subjects’ search.

In Fig. 3, sampling amounts for each subject and in each period are plotted across the two treatments. The spike in DFE-HT occurs right when the participant has filled this sampling-round’s history table. We infer from this that the history table’s maximum capacity (always set at 57 draws) was a very potent cue for search termination in *DFE-HT*. In its absence, participants’ search-effort followed a more normal-like distribution.

*Variance and experienced variability*

We first examine the effect of experienced-outcome^7^ variability (variability for short). Following Lejarraga et al. (2012) we distinguish between: positive variability if someone sampled more than one type of cards in a deck and no variability otherwise. Comparing the means of these two groups we verify that experiencing positive variability correlates positively with higher amounts of sampling. Specifically, sampling amount for positive variability averaged 33.5 draws per lottery while that for no variability 19.5 (*p* value <0.01, two-sided MW test).

We turn next to the relation between sampling amount and a lottery’s variance where we compute averages of sampling amount for each level of variance and examine how the two correlate in each sampling treatment. Subjects only sampled binary lotteries and hence variance was always strictly positive. As mentioned earlier, low variance is associated with rare events. For example a binary lottery offering 1 with probability *p* and 0 otherwise has variance: $p(1-p)$ which is maximized when $p=1/2$, i.e. when the rarity of the rarer event is minimized.

**Fig. 4:**
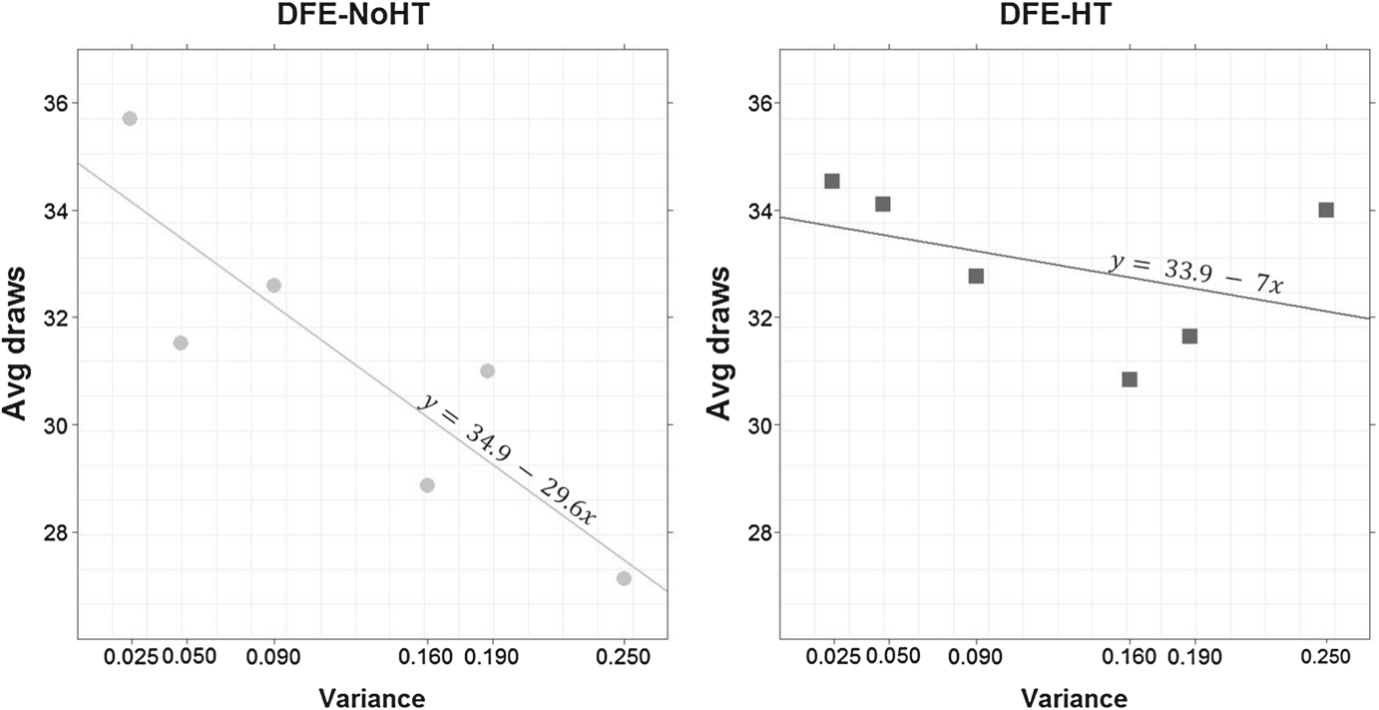
Average sampling amount over levels of variance. *Points* represent average sampling—across all subjects—for different levels of variance in* DFE-NoHT* (*left panel*) and *DFE-HT* (*right panel*). The *solid straight lines* have been estimated by OLS at the aggregate level. Lotteries like: $(x,E_\mathrm{p};y)$ and $(x, E_{1-p};y)$ are indistinguishable during sampling and were pooled together. Lotteries and hence levels of variance were randomized for each subject and so this effect is independent of time period

In both DFE treatments variance correlates negatively with search effort. Interestingly, this correlation is significant in DFE-NoHT (Spearman’s $\rho =-0.89$, *p* value $=$ 0.03) but not in DFE-HT ($\rho =-0.6$, *p* value $=$ 0.24). Figure 4 displays this information.

Individual level analysis corroborates this finding. We estimate slopes for each subject from a simple linear regression, where average sampling over all rounds is regressed on levels of variance (a slope similar to the one in Fig. 4 but for each individual). Although average slopes are negative in both treatments (DFE-NoHT: $-29.61$ vs. DFE-HT: $-6.98$), only in DFE-NoHT this coefficient is significantly smaller than 0 (*p* value <0.01 for DFE-NoHT and *p* value $=$0.146 for DFE-HT, one-sided MW tests). Moreover, the slope is steeper in DFE-NoHT than in DFE-HT (*p* value $=$ 0.043, one-sided MW-test). Estimating rank correlation coefficients instead of slopes replicates this analysis. In both treatments the average correlation is negative ( DFE-NoHT: $-0.219$, DFE-HT: $-0.053$) but only in DFE-NoHT this coefficient is significantly smaller than 0 (*p* value <0.01 for DFE-NoHT and *p* value $=$ 0.259 for DFE-HT, one-sided MW-tests). Since lotteries with rarer events are associated with lower variance, we can state the following result:

#### Result 1

Decks containing rarer events instigate higher search-effort. The history table partially mitigates this effect.

Result 1 runs opposite to Lejarraga et al.’s hypothesis that experienced variability causes higher amount of sampling. We return to this point in the Discussion.

*Time periods*

**Fig. 5:**
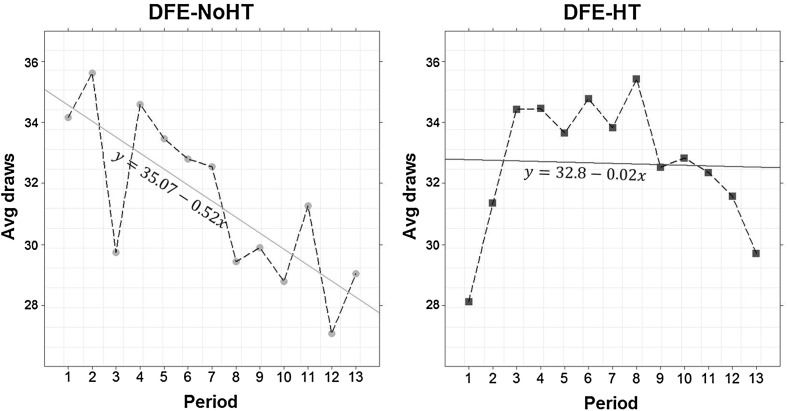
Average sampling amount over periods. Points represent average sampling—across all subjects—for different time-periods in DFE-NoHT (*left panel*) and *DFE-HT* (*right panel*). Arguably the OLS at the aggregate level that is used to plot the *solid straight lines* is not informative for the DFE-HT treatment where the shape is inverted U

Figure 5 plots average sampling amount over time. We see that in DFE-NoHT there is a clear negative trend: subject possibly get tired of sampling over time. In DFE-HT the pattern is inverted U-shaped. It is possible that subjects realize the benefits of the history table after the end of the first sampling round and adjust their strategy to collecting larger samples. After this original upwards-adjustment, sampling amount stabilizes at a high level until it eventually decays in the last periods.

We detect a significant negative time trend in search effort in DFE-NoHT ($\rho =-0.78$; *p* value <0.01). We found no significant such trend in DFE-HT ($\rho =-0.13$, *p* value $=$ 0.66). This is most likely due to the fact that with the exception of the first and last periods, sampling amount remained relatively unaffected by time in DFE-HT. Comparing the variances of average sampling amounts from periods 2 to 12, we find that the variance in DFE-HT (1.81) is smaller than the one in DFE-NoHT (6.97). Levene’s test for variance equality shows that the two variances are significantly different (*p* value $=$ 0.028). When we look only at the second half of the time periods, we verify that eventually time affected subjects in DFE-HT too (Spearman’s $\rho =-0.90$ , *p* value <0.01). In summary:

#### Result 2

Sampling amount diminishes over time. This effect is less prominent in DFE-HT.

Slope and rank correlation analysis at the individual level verify this result. For brevity we report only rank correlation coefficients. For DFE-NoHT this coefficient was on average significantly smaller than 0 ($\rho =-0.14$, *p* value $=$ 0.033, one-sided MW-test) and significantly smaller than the average for DFE-HT (*p* value $=$ 0.033, one-sided MW-test). The average rank correlation coefficient for DFE-HT is not significantly different than 0 ($\rho =0.04$, *p* value $=$ 0.492) but once again, when we focus on the second half of the periods, it becomes significantly (albeit weakly) negative ($\rho =-0.127$, *p* value $=$ 0.051, one-sided MW-test).

*Memory*

We examine whether the history table boosted search effort across the two treatments. First, we find that the median sampling amount across both treatments was 30, which is unusually high. This number was $7\pm 2$ in most studies in the sampling paradigm (Hertwig and Pleskac 2010) and between 15 and 21 in AHP. Consequently, in the current study subjects did not sample both types of cards in only $10\%$ of the cases ($9\%$ in DFE-HT, $11\%$ in DFE-NoHT). In Hertwig et al. (2004) that number is $44\%$. Nevertheless, sampling levels were not significantly different between the two DFE treatments. The median number of draws for DFE-HT was 30 while that for DFE-NoHT was 28 (*p* value $=$ 0.158, two-sided MW-test). Moreover, the forward digit span task, which served as our proxy for working memory, did not correlate with sampling amount in either treatment ($\rho =0.13$, *p* value $=$ 0.41 and $\rho =0.22$, *p* value $=$ 0.15 for DFE-NoHT and DFE-HT respectively).

### Choices and preferences

#### The DE gap in choice

**Fig. 6:**
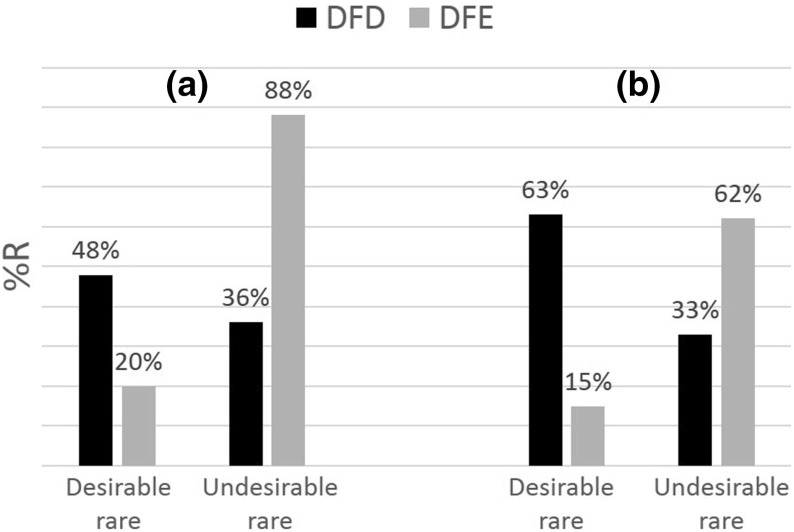
Choice patterns in early DE gap studies. Percentage choosing ‘Risky’ over ‘Safe’ across studies (**a**, **b**), treatments (DFD and DFE) and decision problems (‘desirable rare’ and ‘undesirable rare’). **a**Hertwig et al. (2004) **b** Hau et al. (2008)/Study 1. *Desirable rare*/${\text {Risky}}=(32, E_{0.1}; 0)$ vs. ${\text {Safe}}=(3,E_{1.0})$. *Undesirable rare*: ${\text {Risky}}=(4, E_{0.8}; 0)$ vs. ${\text {Safe}}=(3, E_{1.0})$

In this section, we examine the DE gap in choice over lotteries without the mediation of a preference model. We first look at the choice patterns reported by two important early studies in this literature: Hertwig et al. (2004) and Hau et al. (2008). These studies share a common set of decision problems where a subject is asked to choose between two options with similar EV but differing variance. We refer to the high variance option as ‘Risky’ and the low variance option as ‘Safe’. To increase comparability with our study we consider only those decision problems that entail non-negative outcomes and where the ‘Safe’ option is a certain outcome (see Appendix/Table 6 for the full list of decision problems). This restricts the analysis to 2 decision problems (from a total of 6) which we then characterize according to the desirability of the rare outcome of the ‘Risky’ option. Decision problems with a rare (un)desirable outcome are referred to as ‘(un)desirable rare’. Let ‘$\%R$’ stand for the percentage with which subjects chose ‘Risky’ over ‘Safe’. Figure 6 plots $\%R$ across treatments in these two studies for ‘desirable rare’ and ‘undesirable rare’.

Table 2 lists the properties of the early DE gap according to the observed choice-patterns. Properties 1 and 2 derive from comparisons between DFD and DFE while Properties 3 and 4 from comparisons within each treatment. Property 1 is that people choose ‘Risky’ over ‘Safe’ more often in DFD than in DFE when the rare outcome is desirable while Property 2 is that the opposite holds true when the rare outcome is undesirable instead. Property 3 is that subjects in DFD choose ‘Risky’ over ‘Safe’ more often when the rare outcome is undesirable than when it is desirable while Property 4 is that this pattern is reversed when subjects make decisions in DFE.

**Table 2:** Properties of the original DE gap in choice

Property $\#$	Choice pattern	Condition
1.	$\%R_\mathrm{DFD}>\%R_\mathrm{DFE}$	Desirable rare
2.	$\%R_\mathrm{DFD}<\%R_\mathrm{DFE}$	Undesirable rare
3.	$\%R_\mathrm{Desirable} >\%R_\mathrm{Undesirable}$	DFD
4.	$\%R_\mathrm{Desirable}<\%R_\mathrm{Undesirable}$	DFE

**Fig. 7:**
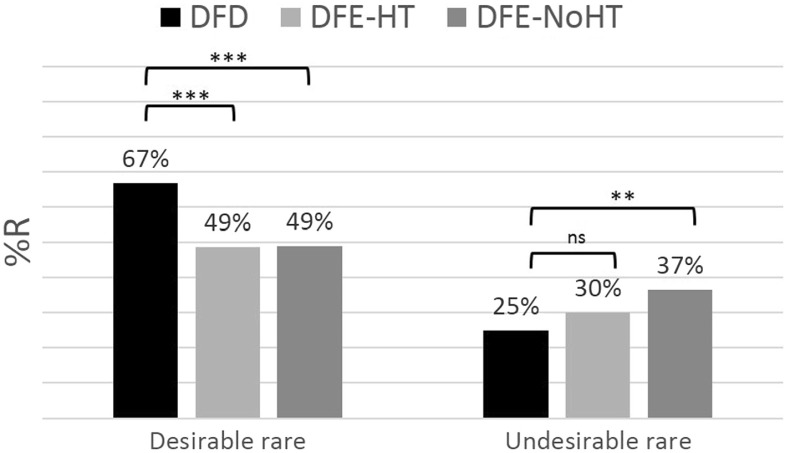
Choice patterns in this study. Percentage choosing ‘Risky’ ($\%R$) over ‘Safe’ in the current study across treatments (DFD, DFE-HT and DFE-NoHT) and types of decision problems (desirable and undesirable rare). ‘Risky’ refers always to the lottery and ‘Safe’ to its expected value. We consider lotteries 8–16 from Table 1 and cluster choices in the following way: *‘Desirable rare’*: Risky $=(16, E_\mathrm{p}; 0)$ for $p\le 0.25$. *‘Undesirable rare’*: Risky $=(16, E_\mathrm{p}; 0)$ for $p \ge 0.75$. *ns* not significant, ***p value < 0.01, **p value < 0.05

Figure 7 plots results from our study using an analysis similar to that summarized in Fig. 6. Recall that although our method relies on lottery-valuations, these valuations take place via repeated choices. For this analysis we use lotteries 8–16 from Table 1). These are the same lotteries which we later use to elicit weighting functions and therefore appropriate to compare the two types of DE gap: that inferred by choice-patterns (sampling paradigm) and that inferred by weighting patterns (AHP). We separate these lotteries into two clusters: those with $p\le 0.25$ and those with $p \ge 0.75$. Decision problems entailing a choice between a lottery with $p\le 0.25$ and its EV are characterised as ‘desirable rare’ since the rare^8^ event is associated with the high outcome (£16). Decision problems entailing a choice between a lottery with $p\ge 0.25$ and its EV are characterised as ‘undesirable rare’ since the rare event is associated with the low outcome (£0). This analysis leaves out only the lottery with the 50–50 distribution where no event can be considered to be rarer than the other.

According to Fig. 7, choice patterns in DFD are significantly different than in DFE-HT and DFE-NoHT for ‘desirable rare’ (*p* value <0.01 for both DFD vs. DFE-HT and DFD vs. DFE-NoHT, two-sided proportion test). For the ‘undesirable rare’, however, only the DFD vs. DFE-NoHT comparison is significant (*p* value $=$ 0.037 for DFD vs. DFE-NoHT and *p* value $=$ 0.384 for DFD vs. DFE-HT, two-sided proportion test). Result 3 summarizes this analysis.

##### Result 3

Both versions of DFE generate a significant DE gap. This gap is smaller in the presence of the history table.

Moreover, comparing the choice patterns in Fig. 7 with Table 2 we verify that 3 out of these 4 properties of the early DE gap hold in this analysis. However, the fact that $\%R$ in DFE is higher in the ‘desirable rare’ than in the ‘undesirable rare’ violates Property 4. With this in mind, we claim that:

##### Result 4

The DE gap we capture in this study is qualitatively similar but not identical to the original phenomenon.

We examine two hypotheses for the low level of $\%R_\mathrm{Undesirable}$ in DFE. First, we consider the possibility that this is due to the asymmetry in the EV of the risky option between early DE gap studies (3.2) and the current one (14.3 on average). Second, we conjecture that the difference is driven by information-asymmetries between the two paradigms: unlike the sampling paradigm, subjects in our study were always informed about the existence of the second outcome. Moreover, due to the higher levels of sampling we recorded, rare events were under-represented less often than in earlier studies.

With respect to the first hypothesis, we examine choices from control lottery: $(4,E_{0.8};0)$ and observe that the pattern is very similar to that in Fig. 7 ($\%R_\mathrm{DFD}=26\%, \%R_\mathrm{DFE-HT}=30\%, \%R_\mathrm{DFE-NoHT}=31\%$; see Appendix/Table7 for details on the choice patterns of all ‘control’ lotteries). For the second hypothesis we repeated the analysis in Fig. 7 but considering only cases in which the probability of the rare event has been under-represented. We see that in this case all 4 properties of the early DE gap hold for the comparison between DFD and DFE-NoHT (but still not for that between DFD and DFE-HT; see Appendix/Fig. 10) and, therefore, conclude that the second hypothesis is more likely to be the explanation behind the violation of Property 4.

One last thing to notice about Fig. 7 is that risk aversion (as inferred by $\%R$) is probability dependent. In DFD subjects seem to be overall risk seeking ($\%R>50\%$) for small gain probabilities (i.e. when the rare event is desirable) but risk averse ($\%R<50\%$) for high gain probabilities (i.e. when the rare event is undesirable). This is in accord with CPT’s fourfold pattern. In DFE, subjects seem to be overall risk neutral ($\%R\simeq 50\%$) for small gain probabilities but risk averse (albeit comparatively less so than in DFD) for high gain probabilities.

#### The DE gap in preferences

We proceed by incorporating in the analysis all iterations of the bisection and extracting a CE for each lottery. We use these CEs to estimate CPT’s components as described under Sect. 2.2.2. We start by comparing utility curvature ($\alpha$) across treatments. Median values in all treatments suggest a near linear utility curvature (Table 3). These values are higher than those reported by AHP ($\alpha =0.79$ for DFD and $\alpha =0.82$ for DFE) as well as than the usual values reported by studies with medium to low awards (slightly less than 1; see Booij et al. 2010). They are nevertheless within the typically reported range (see Murad et al. 2015; Epper et al. 2011 for values of $\alpha$ slightly higher than 1). By classifying subjects according to utility curvature ($\alpha <0.9$ as concave, $\alpha \in [0.9,1.1]$ as linear and $\alpha >1.1$ as convex), we see that overall most of the subjects (57%) are best characterized by a utility function that is either concave or linear rather than convex (see Appendix/Table 8 for more details). There were no significant differences between $\alpha$’s across treatments (*p* value $=$ 0.77, Kruskal–Wallis).

**Table 3:** Median estimates of $(\alpha , \delta , \gamma )$

Treatment	Utility curvature ($\alpha$)	Weighting elevation ($\delta$)	Weighting curvature ($\gamma$)
DFD	1.06 (0.37)	0.53 (0.13)	0.49 (0.12)
DFE-HT	1.06 (0.35)	0.48 (0.10)	0.52 (0.07)
DFE-NoHT	1.02 (0.35)	0.44 (0.11)	0.67 (0.08)

For DFE treatments, the $\delta$’s and $\gamma$’s are estimated according to experienced probabilities. Median standard errors from the estimation procedure are reported in parentheses. Overall, parameters were equally dispersed across treatments; equality of variance was never rejected (*p* value $=$ 0.199 for $\alpha$, 0.722 for $\gamma$ and 0.804 for $\delta$, Levene’s tests). Interquartile ranges were: [0.83–1.49] for $\alpha$, [0.20–0.91] for $\delta$ and [0.37–0.87] for $\gamma$

Having estimated $\alpha$, we can use Eq. 5 to calculate decision weights for each individual. Treatment-level weighting functions can be obtained either by aggregating weights across subjects for each level of probability (non-parametric analysis) or by fitting the parameters from Eq. 6 for each subject and aggregating $(\gamma ,\delta )$ across all subjects (parametric-analysis).^9^ We begin with the latter.

*Parametric analysis*

Kruskal–Wallis tests detect significant differences between $\gamma$-values across the three treatments (*p* value $=$ 0.038) but not for $\delta$-values (*p* value $=$ 0.501). Focusing on $\gamma$’s, the difference between $\gamma _{_\mathrm{DFD}}$ and $\gamma _{_\mathrm{NoHT}}$ is significant (*p* value $=$ 0.015, two-sided MW-test) while that between $\gamma _{_\mathrm{DFD}}$ and $\gamma _{_\mathrm{HT}}$ only weakly so (*p* value $=$ 0.065, two-sided MW-test). Moreover, the hypothesis that $\gamma _{_\mathrm{NoHT}}=\gamma _{_\mathrm{HT}}$ cannot be rejected ( *p* value $=$ 0.485, two-sided MW test).

**Fig. 8:**
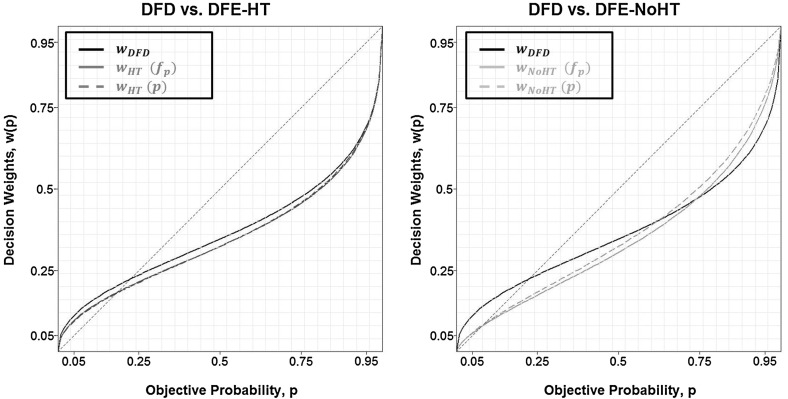
Comparison of parametric weighting functions between DFE and DFD. For DFE, *dashed lines* are estimated according to true probabilities (*p*) while *solid lines* are based on experienced probabilities ($f_\mathrm{p}$)

**Fig. 10:**
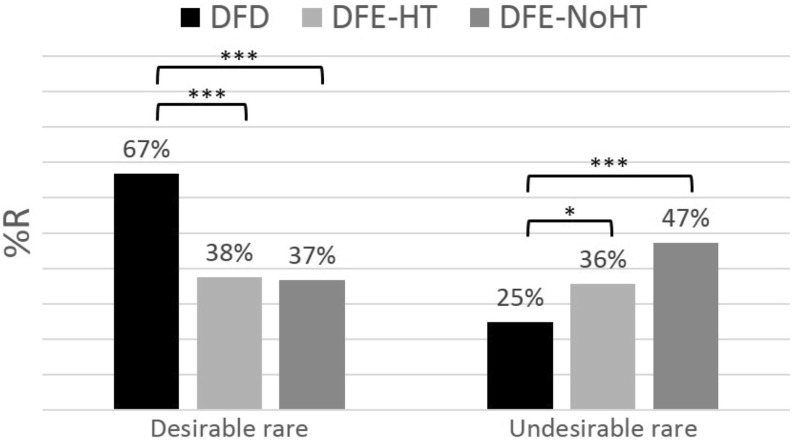
Choice patterns in cases where rare events have been under-represented. ***p value <0.01, *p value <0.10

Figure 8 plots differences in weighting between description and the two versions of experience: with (left panel) and without (right panel) a history table. The proximity between the experienced-based parameter estimates (solid lines) and objective-based such estimates (dashed lines), holds testament to the high amount of sampling which brought experienced and objective probabilities very close. In fact, with the exception of $p=0.975$ for DFE-HT, we were never able to reject the hypothesis that $f_\mathrm{p}=p$ (see Appendix/Table 5 for details). A corollary to this is that the role of sampling bias was—at least at the aggregate level—quite limited.

Unlike what the ‘underweighting hypothesis’ would have predicted, Fig. 8 suggests that the common inverse S-shaped weighting function accommodates well DFD as well as both DFE treatments. Moreover, the relation between $w_{\mathrm{DFD}}$ and both versions of $w_{\mathrm{DFE}}$ provides little support for the ‘ambiguity aversion hypothesis’ according to which $w_{\mathrm{DFE}}$ should lie beneath $w_{\mathrm{DFD}}$ throughout the probability interval. Although this is true for small to medium values of *p*, the pattern reverses for high values of *p* (this is arguably clearer in the case of DFE-NoHT where the turning point occurs somewhere in $p\in [0.6,0.8])$. Keeping in mind that rare events are located near the edges of the probability interval (desirable rare events close to $p=0$ and undesirable rare events close to $p=1$), we can summarize Fig. 8’s pattern as follows:

##### Result 5

The ‘relative underweighting hypothesis’: Subjects overweight rare events in DFD and in DFE; this overweighting is less pronounced in DFE.

At the individual level, we categorize the curvature of weighting functions as ‘inverse-S’ when $\gamma < 0.9$, as ‘S-shaped’ when $\gamma >1.1$ and as ‘no curvature’ when $\gamma \in [0.9,1.1]$. There are approximately twice as many subjects compatible with an ‘S-shaped’ weighting function in DFE (DFE-HT: 10 subjects, DFE-NoHT: 9 subjects) as in DFD (5 subjects). Interestingly, most of these S-shaped curves stem from subjects who sampled less than the median amount of that treatment: $60\%$ in DFE-HT and $89\%$ in DFE-NoHT (see more details of this classification in Appendix/Table 8). A rank correlation test between sampling behaviour (1 if someone sampled less or equal to the median amount and 0 if more) and curvature of the weighting function (1 if $\gamma >1.1$ and 0 otherwise) verifies that there is a significant correlation between the two ($\rho =0.318$, *p* value<0.01).^10^ No such correlation was detected for similar classifications of $\delta$ ($\rho =-0.038$, *p* value $=$ 0.736).

##### Result 6

S-shaped weighting curves are more common to subjects who sample less.

Result 6 may be very useful in explaining why we find so little support of the ‘underweighting hypothesis’; we return to this point in the discussion section.

*Non-parametric analysis*

Table 4 reports average decision weights—computed according to experienced probabilities ($f_\mathrm{p}$)—across individuals according to probability level and treatment.^11^ Qualitatively the non-parametric analysis corroborates Result 5: aggregate decision weights point to inverse S-shaped weighting functions in all treatments with a cross-over point in the vicinity of $p=0.25$. This is supported by statistical analysis comparing decision weights with the diagonal (see last three columns of Table 4). Moreover, this overweighting appears to be partially mitigated for rare events: $w_{_\mathrm{DFE}}<w_{_\mathrm{DFD}}$ for $p<0.25$ and ($1- w_{_\mathrm{DFE}})<(1-w_{_\mathrm{DFD}})$ for $p>0.75$. Statistical analysis, however, warrants a note of caution regarding the last assertion. A $3\times 9$ ANOVA does not detect any significant differences between the 3 treatments (*p* value $=$ 0.412).^12^

**Table 4:** Non-parametric decision weights (averages)

Probability	DFD	DFE-HT	DFE-NoHT	DFD vs. p	DFE-HT vs. p	DFE-NoHT vs. p
*p*	$w_{_\mathrm{DFD}}$	$w_{_\mathrm{HT}}$	$w_{_\mathrm{NoHT}}$	$t_{39}$	$t_{40}$	$t_{39}$
0.025	0.16	0.11	0.09	$$4.58^{***}$$	$$2.38^{**}$$	$$2.35^{**}$$
0.05	0.18	0.16	0.12	$$4.24^{***}$$	$$2.99^{***}$$	$$2.82^{***}$$
0.10	0.20	0.18	0.15	$$3.23^{***}$$	$$2.36^{**}$$	$$2.12^{**}$$
0.25	0.25	0.21	0.21	$-0.09^{\mathrm{ns}}$	$-1.43^{\mathrm{ns}}$	$1.44^{\mathrm{ns}}$
0.50	0.36	0.30	0.31	$$-3.64^{***}$$	$$-5.52^{***}$$	$$-5.52^{***}$$
0.75	0.49	0.47	0.49	$$-6.41^{***}$$	$$-6.31^{***}$$	$$-6.31^{***}$$
0.90	0.63	0.65	0.65	$$-5.48^{***}$$	$$-5.20^{***}$$	$$-5.19^{***}$$
0.95	0.60	0.69	0.69	$$-7.11^{***}$$	$$-5.07^{***}$$	$$-5.02^{***}$$
0.975	0.71	0.72	0.74	$$-5.43^{***}$$	$$-4.07^{***}$$	$$-4.07^{***}$$

Columns 2–4: average decision weights for each level of probability (*p*). For brevity, only weights that have been estimated according to experienced probabilities ($f_\mathrm{p}$) are reported.

Columns 5–7: two-sided *t*-statistics for the comparison with the identity line. With the exception of $p=0.25$, low probabilities are significantly overweighted (see ‘$+$’ sign on *t*-statistic) and medium to high probabilities significantly underweighted (see ‘−’ sign on *t*-statistic). Two sided MW-tests confirm this analysis

ns not significant

$$^{***}\, \mathrm{p~value} <0.01$$

$$^{**} \,\mathrm{p~value} <0.05$$

#### Recency effects

We explore whether events experienced towards the end of the sampling process influenced choices more than events that were sampled in the beginning. To this end we folow AHP’s approach^13^ and compare absolute differences between revealed and experienced probabilities as inferred from the first and second halves of each sampling round. Revealed probabilities are estimates of $P(E_\mathrm{p})$, the likelihood assigned by the subject to event $E_\mathrm{p}$ (see expression (2) in Sect. 2.2.1). These estimates derive from the estimated inverse images: $w_\sigma ^{-1}[w_\sigma (f_\mathrm{p})]$. Had recency effects been present, we would expect the $f_\mathrm{p}$ of the second half of the sampling process to be closer to $P(E_\mathrm{p})$ estimates. Notwithstanding, a $2 \times 9$ ANOVA with repeated measures for the first and second half did not detect significant asymmetries between the early and the later observations of the sampling process (*p* value $=$ 0.73 for DFE-NoHT and 0.64 for DFE-HT).^14^ We thus conclude that there were no recency effects.

## Discussion

*Variance vs. variability*

We began by exploring the effect on sampling amount of two related concepts, experienced event variability and a lottery’s variance. We verify that experienced variability correlates with higher levels of sampling. Does that mean, however, that experiencing variability *causes* subjects to sample more as Lejarraga et al. (2012) have claimed? Or is it rather that high levels of sampling lead subjects to sample more than one event? To clarify the direction of causality we examined the role of variance which is a proxy for experienced variability: lotteries with higher variance are more likely to generate experienced variability. At the same time, unlike experienced variability, variance is a structural property of the lottery and thus cannot be affected by the amount of sampling. In our setting, low variance is associated with rarer events. Therefore, if experienced variability causes higher levels of sampling, we would expect high-variance lotteries to be associated with higher levels of sampling. Instead, Fig. 4 and Result 1 point to the opposite: subjects sample more from lotteries with low variance, or equivalently, lotteries containing rarer events. According to a property of the binomial distribution, rare events tend to be revealed later on during search. Consequently, Result 1 has more in common with Mehlhorn et al.’s (2014) suggestion that it is anticipated rather than experienced variability that instigates higher levels of sampling.

*Does the history table crowd out attention from the sampling process?*

As Result 1 suggests, the increased sensitivity towards rare events was attenuated in the anticipation of the history table. Result 2, highlights another such search-policy rigidity in DFE-HT. Unlike the clear negative time-trend in DFE-NoHT, average sampling in DFE-HT has a significantly less steep decline. In fact, excluding first and last periods, average sampling remained relatively stable in DFE-HT (we observed significantly lower variance of average sampling compared to DFE-NoHT during these periods). One possible overarching explanation for these findings is that the anticipation of the history table makes cues unrelated to it less salient. Figure 3 can perhaps be interpreted along these lines. The frequency with which subjects in *DFE-HT* chose to collect a sample just equal to the table’s maximum capacity, corroborates the hypothesis that cues such as time and variance were overriden by that of filling up the history table.

*Memory limits*

Taking into account their elusive nature we chose to approach the potential effects of memory bounds from three different angles. First, we asked whether alleviating the cognitive load of memorizing via the history table can boost search effort. Second, we examined whether individual idiosyncratic memory capacity correlates with the size of drawn samples. Finally, we examined whether later observations exert more influence on final decisions when compared to earlier ones. Despite this multidimensional approach we were unable to detect a clear effect in all three accounts. Subjects’ sample size did not vary significantly between DFE-HT and DFE-NoHT nor did it correlate with the forward digit span task. Finally, we find no evidence for recency effects.

Given the intuitive appeal of the role of memory bounds this absence of effects may seem counter-intuitive. This impression is only strengthened by the fact that in our study samples were unusually high, which should have amplified the impact of the role of memory. However, these results add to an increasing amount of evidence that challenges the importance of memory bounds (e.g. Rakow et al. 2008; Wulff et al. 2016 for a relevant discussion). To this end, we welcome studies that seek to understand how decisions are informed by exploring mechanisms beyond plain memorisation.

*Why so much sampling?*

Subjects in both versions of our DFE treatments were much more eager to explore options than what has commonly been reported. One explanation for this search ‘explosion’ relates to the absence of waiting time between two consecutive draws. In our experiment subjects were able to regulate the time the card remains on their screen. On the one hand, this feature increased clicking effort as subjects had to click twice—instead of only once which is more typical—before observing a new card: first to replace the previously drawn card and then to sample a new one. On the other hand, this adaptation made subjects’ role during exploration more active as well as made the sampling process quicker—should subjects choose to click fast enough. It has been argued that in DFE, subjects are the ‘masters of their information search’ (Hills and Hertwig 2010) and in this sense this study’s framework takes this exploration-ownership one step further. Perhaps the more subjects relate to the role of an actor instead of that of an observer, the more encouraged they feel to explore further. A more prosaic explanation would be that the cost of clicking twice is a small price to pay for removing waiting time and, therefore, our intervention simply reduced the opportunity cost of sampling.

*The DE gap across different elicitation methods*

The differences between AHP’s methodology (which this study adopts) with that of the sampling paradigm in inferring a DE gap, have raised concerns regarding the compatibility of the findings within these two approaches. Results 3 and 4 are reassuring in that respect. Result 3 shows that our method can detect a significant DE gap even without the mediation of a preference model, by focusing only on choice patterns. These choices are elicited from the first iteration of the bisection method which entails a choice between a risky and a safe option of equal EV; a setting very similar to that in early DE gap studies. Moreover, according to Result 4, this DE gap is qualitatively similar to that elicited in the sampling paradigm. Just as in Hertwig et al. (2004), subjects in our study chose the risky option more frequently in DFD than in DFE when rare events were associated with desirable outcomes while the opposite was true when the outcomes were undesirable. However, unlike in the early DE gap studies, subjects in our DFE treatments were overly hesitant in choosing ‘Risky’ in ‘undesirable rare’ decision problems. We discuss two possible explanations for this.

First, the fact that subjects knew about the existence of the (rare) undesirable outcome might have contributed to their hesitation of choosing ‘Risky’. This is in accord with the ‘mere presentation effect’ discussed in Erev et al. (2008). Unlike the sampling paradigm where if this outcome was never sampled subjects might had never inferred its existence, AHP’s method requires that subjects eventually found out about this outcome. Moreover, the fact that subjects in our study sampled a lot and were overall very well informed about the likelihood of the undesirable outcome might have amplified this effect. Indeed, when we look only in samples where this probability was under-represented we see that subjects become more willing to take the risky option in such ‘undesirable rare’ decision problems. Second, we consider the discrepancy between the EV of lotteries under consideration. In earlier studies, subjects typically faced lotteries with an EV of approximately £3 (or less). In our study that EV was somewhere between £12 and £15.6 which could have made subjects more hesitant to reject the safe option. Given, however, that our analysis of the control lottery: $(4,E_{0.8}; 0)$ replicated this unusually high hesitation we believe that our first hypothesis is more likely to be the case.

*The relative underweighting hypothesis*

Our elicited weighting patterns provided little support for both the ‘underweighting’ and the ‘ambiguity aversion’ hypotheses. With respect to the first, our data in all treatments reveal—at the aggregate level—an inverse S-shaped weighting function which prescribes overweighting instead of underweighting of rare events. Moreover, unlike the second hypothesis, DFE-elicited weighting curves do not lie entirely beneath that elicited in DFD. Instead, our pattern seems to fit best under a third hypothesis that can be interpreted as a modest version of the underweighting one. The ‘relative underweighting hypothesis’ as summarized by Result 5 posits that although subjects overweight rare events in DFE, they do it less so than in DFD.

Regarding the discord with the ‘underweighting hypothesis’, Result 5 is not entirely surprising. Over the last few years, an increasing amount of studies have also failed to detect a S-shaped weighting curve, irrespective of the elicitation method they followed (e.g. AHP, Aydogan and Gao 2016; Glöckner et al. 2016). One possible explanation for the absence of a S-shaped pattern in our DFE treatments is related to the high levels of sampling amount we recorded. Indeed, Result 6 seems to point in that direction as S-shaped weighting functions are prevalent among subjects who sample less. This is not surprising: subjects who do not sample enough are more likely to under-represent, and thus underweight rare events. It is, therefore, plausible that if our levels of sampling had been significantly lower, we might have seen more evidence for the ‘underweighting hypothesis’.

With respect to the disagreement with the ‘ambiguity aversion hypothesis’ we suggest the following explanation. The fact that subjects in our study collected larger samples than those in AHP might have affected their confidence during the evaluation of the lotteries. It is true that subjects in DFE can never be entirely certain regarding the underlying probability distribution. Nevertheless, richer information sets—such as the ones collected in our study—could have increased their confidence about those likelihoods and consequently reduced the associated ambiguity aversion.

*Does the history table bridge the DE gap?*

Lastly, we turn to a comparison of the DE gap between the two versions of experience that caused it. Result 3 suggests that although the gap is significant in both cases, its size is not symmetric. Specifically, our choice patterns reveal a bigger DE gap between DFD and DFE-NoHT. This asymmetry is corroborated by the weighting function comparison $-w_\mathrm{DFD}$ and $w_\mathrm{HT}$ are ‘closer’ than $w_\mathrm{DFD}$ and $w_\mathrm{NoHT}$—as well as by the resistance of DFE-HT to ‘conform’ to all 4 properties of the original DE gap, even when we focus on under-represented probabilities.

To the extent that the analogical display of previously sampled events in DFE-HT has a similar ‘descriptive’ effect to the numerical summaries of uncertainty in DFD, this result should not come as a surprise. We interpret this ‘bridging’ of the gap as evidence that the DE gap should not be seen as a dichotomy but rather as a continuum over different levels of uncertainty.

## Conclusion

We conduct a lab-experiment and examine how people search for information about uncertainty and how this search influences their ensuing risky choices. We find that besides the properties of the risky options at hand, the environment in which these options are presented and evaluated is also important. With respect to search patterns in DFE, we show that a lottery’s variance is negatively correlated with sampling amount which in this context means that people sample more from options with rarer events. We also find that sampling amount decreases over time periods. Both of these findings become less salient after the introduction of a history table which records and displays previously sampled outcomes at the time of the lottery evaluation. The cue that stands out in that case is the maximum capacity of that table. Moreover, our examination of the role of memory in sampling suggests that memory bounds were not very influential on search policies.

With respect to choices and preferences we compare responses between two variations of DFE: with (DFE-HT) and without a history table (DFE-NoHT) and compare them with those elicited from a standard version of DFD. Both of these comparisons generate a significant DE gap which is mitigated, however, by the inclusion of the history table. We interpret these choices through the CPT preference model by eliciting risk curvature (parametrically) and weighting functions (both parametrically and non-parametrically) at the individual level. Although utility curvature does not differ across treatments, the shape of decision weighting functions does. In our version of the DE gap in weighting, subjects in DFE overweight rare events but less so than in DFD. We show that the absence of under-weighting in DFE can partially be explained by the unusually high levels of sampling observed in our study.

Finally, we report a measure that allows us to compare the type of gap found in studies using valuation methods—like this one—with the type of gap elicited in studies that use choice methods. We show that the phenomenon is qualitatively similar but not identical between the two methods.

## Figures and Tables

**Fig. 9:**
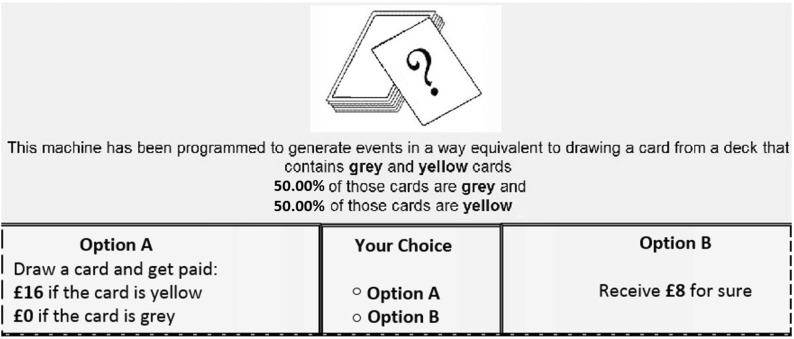
Instance from DFD’s information-evaluation stage. Information about the deck and lottery evaluation take place in the same screen in DFD

**Fig. 11:**
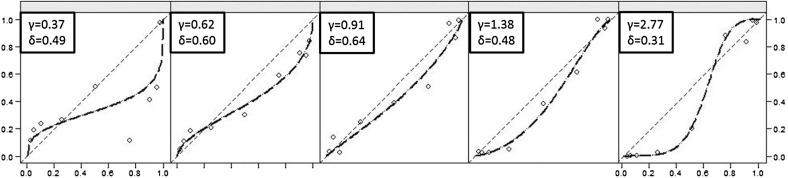
Examples of decision weights elicitation. Examples of non-parametric (*circles*) and parametric (*curves*) weighting functions. From *left* to *right*: increasing values of $\gamma$ for a relatively small range of $\delta$-values. There are two ways of constructing an aggregate weighting function from these five examples. The parametric approach entails aggregating across $\gamma$ - and $\delta$- values while according to the non-parametric one, we would aggregate across the decision weights for each level of probability

**Fig. 12:**
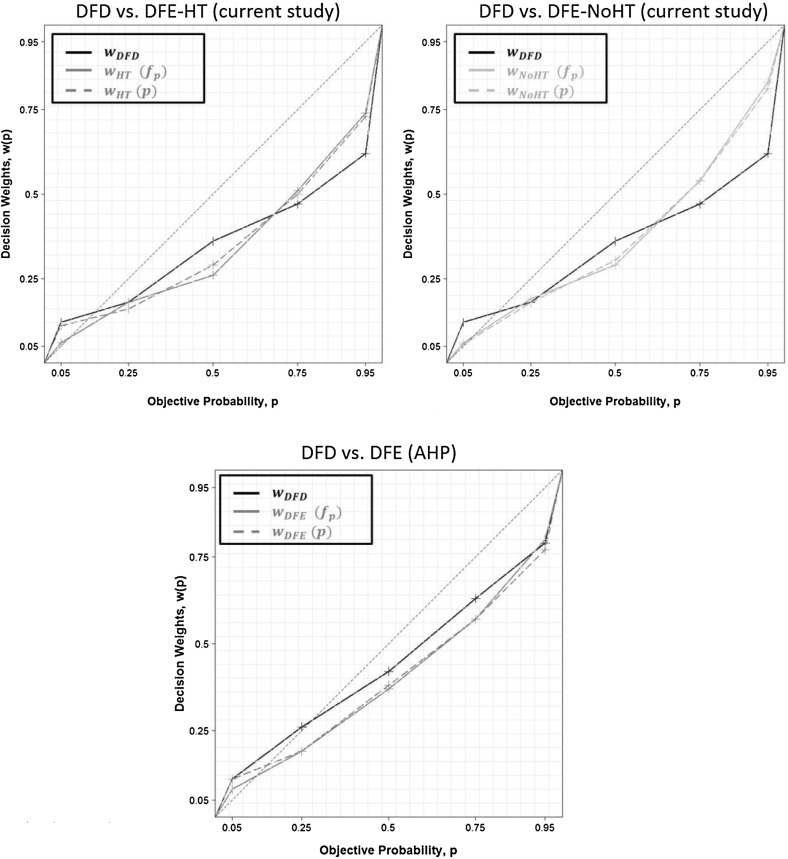
Non-parametric weighting functions in the current study and in AHP. Plotting values from Table 9. *Top row* current study; *bottom row* AHP. For DFE, *dashed lines* are estimated according to objective probabilities (*p*) while *solid lines* according to experienced probabilities ($f_\mathrm{p}$). Only probability targets included in AHP are plotted. This excludes observations at $p\in \{0.025,0.10, 0.90, 0.975\}$ from this study

## Appendix

### Screen for DFD

See Fig. 9.

### Choice patterns

See Fig. 10 and Tables 5, 6, 7.

**Table 5:** Median experienced probabilities for DFE-HT and DFE-NoHT

*p*	Ep	
	DFE-HT	DFE-NoHT
0.025	0.030	0.025
0.050	0.069	0.052
0.100	0.089	0.103
0.250	0.231	0.230
0.500	0.521	0.504
0.750	0.753	0.752
0.900	0.915	0.889
0.950	0.951	0.947
0.975	0.983	0.977

With the exception of *p* $=$ 0.975 for DFE-HT (*p* value <0.01, two-sided MW-test), we are never able to reject the hypothesis that $p=E_\mathrm{p}$

**Table 6:** Decision set from Hertwig et al. (2004)

Decision	Lotteries	
problem	Risky	Safe
1	$(4,E_{0.8};0)$	$(3,E_{1.0})$
2	$(4,E_{0.2};0)$	$(3,E_{0.25};0)$
3	$(-32, E_{0.1};0)$	$(-3,E_{1.0})$
4	$(-4, E_{0.8};0)$	$(-3,E_{1.0})$
5	$(32,E_{0.1};0)$	$(3,E_{1.0})$
6	$(32,E_{0.025};0)$	$(3,E_{0.25};0)$

**Table 7:** Choice patterns in ‘control’ lotteries

Decision	Lotteries		$\%R$		
problem	Risky	Safe	DFD (%)	DFE-HT (%)	DFE-NoHT (%)
*A*	$(4,E_{0.8};0)$	$(3.2,E_{1.0})$	26	30	31
*B*	$(4,E_{0.2};0)$	$(0.8,E_{1.0})$	69	73	53
*C*	$(3,E_{0.25};0)$	$(0.75,E_{1.0})$	67	65	67

Risky options in this table were included as ‘control’ tasks due to their similarity with some of the commonly used problems in the sampling paradigm (see Table 6). For example decision problem 2 in Table 6 corresponds to a choice between the risky option in *B* and the risky option in *C* from this table. Since these lotteries could only be evaluated separately in this study, we can compare choice patterns only indirectly by comparing $\%R$ across problems *B* and *C*. According to early DE gap, $\%R$ should be higher in *B* than in *C* for DFD while the opposite must be true for DFE. This pattern is verified in the comparison between DFD and DFE-NoHT but not between DFD and DFE-HT. Moreover, $\%R$ should be higher in DFE than in DFD for problem *A*. This is indeed the case for both DFE-NoHT and DFE-HT. All of the aforementioned differences are relatively small and not statistically significant

### Individual analysis

See Fig. 11 and Table 8.

**Table 8:** Classification of subjects according to the curvature of utlity and weighting functions

	Utility function			Weighting function		
	Concave (%)	Linear (%)	Convex (%)	Inverse S-shaped (%)	No curvature (%)	S-shaped (%)
	$\alpha <0.9$	$\alpha \in [0.9,1.1]$	$\alpha >1.1$	$\gamma <0.9$	$\gamma \in [0.9,1.1]$	$\gamma >1.1$
DFD	33.3	25.6	41.0	82.1	5.1	12.8
DFE-HT	35.0	20.0	45.0	62.5	12.5	25.0
DFE-NoHT	35.9	20.5	43.6	69.2	7.7	23.1

Classification of $\gamma$’s was made according to decision weights calculated based on objective probabilities

### Non-parametric analysis

See Fig. 12 and Table 9.

**Table 9:** Median decision weights in this study and in AHP

*p*	Current			AHP	
	DFD	DFE-HT	DFE-NoHT	DFD	DFE
0.05	0.12	0.06	0.06	0.11	0.08
0.25	0.18	0.18	0.18	0.26	0.19
0.50	0.36	0.26	0.30	0.42	0.37
0.75	0.47	0.51	0.54	0.63	0.57
0.95	0.62	0.74	0.81	0.79	0.80

## Footnotes

1 It is a convention within this literature to refer to events occurring with $p\le 0.20$ as ‘rare’.

2
Hau et al. (2009) use a similar recording device but in their “decisions from records” treatment subjects were only allowed to sample a fixed amount of cards and hence its influence to search cannot be inferred. In our framework subjects can choose instead how much they want to sample.

3 This is a difference with AHP’s study which uses a within-subjects design where subjects always made description-based decisions prior to experience-based ones.

4 This task was not monetarily incentivised.

5 Restricting analysis to gains reduces CPT to the Rank Dependent Utility model (Quiggin 1982).

6 Similarly to AHP, decision weights based on $E_\mathrm{p}$ for probability targets that were not represented in subjects’ samples, were obtained by a linear interpolation of the weighting function at the individual level.

7 In our study this phenomenon is more accurately described as experienced *event* variability. Outcomes refer to monetary consequences while in our sampling stage subjects sampled events which were only later assigned to outcomes.

8 We replicate our results when considering stricter thresholds for rare events such as $p<0.25$ or $p<0.10$.

9 See Appendix 6.3 for a demonstration of this process at the individual level.

10 This result is robust for different classifications of S-shaped curves such as with $\gamma >1$. We used $\gamma$’s based on decision weights from objective probabilities. The reason we did not use weights corrected for $f_\mathrm{p}$ was so that we capture the effect of mis-representing objective probabilities (*p*) in low sampling cases. When we perform the same analysis adjusted for $f_\mathrm{p}$ we find that the correlation is reduced but still significant ($\rho =0.245$, *p* value $=$ 0.029).

11 See Appendix/Table 9 for a comparison of the median decision weights between this study and AHP)

12 Similarly, conducting two-sided MW-tests with Bonferroni corrections, we can never reject the hypothesis that decision weights are equal between DFD and DFE-HT nor between DFD and DFE-NoHT for any level of *p*.

13 See AHP pp. 1890 for more details on this method.

14 Conducting two-sided MW-tests for each level of *p* with Bonferroni corrections corroborate this.
